# Antibacterial Activity of the Alkaloid-Enriched Extract from *Prosopis juliflora* Pods and Its Influence on *in Vitro* Ruminal Digestion

**DOI:** 10.3390/ijms14048496

**Published:** 2013-04-17

**Authors:** Edilene T. dos Santos, Mara Lúcia A. Pereira, Camilla Flávia P.G. da Silva, Lourdes C. Souza-Neta, Regina Geris, Dirceu Martins, Antônio Euzébio G. Santana, Luiz Cláudio A. Barbosa, Herymá Giovane O. Silva, Giovana C. Freitas, Mauro P. Figueiredo, Fernando F. de Oliveira, Ronan Batista

**Affiliations:** 1 Department of Instrumental and Basic Studies, State University of Southeast of Bahia, BR 415, Km 03, s/n, 45.700-000 Itapetinga, Bahia, Brazil; E-Mails: edilenets@yahoo.com.br (E.T.S.); marauesb@yahoo.com.br (M.L.A.P.); kmillaf@yahoo.com.br (C.F.P.G.S.); heryma@gmail.com (H.G.O.S.); 2 Department of Exact and Earth Sciences, University of Bahia, Rua Silveira Martins, 2555, Cabula, 41150-000 Salvador, Bahia, Brazil; E-Mail: lourdes-neta@ig.com.br; 3 Chemistry Institute, Federal University of Bahia, Rua Barão de Geremoabo, s/n, Ondina, 40170-290 Salvador, Bahia, Brazil; E-Mails: rmgeris@ufba.br (R.G.); dirceum@ufba.br (D.M.); 4 Chemistry and Biotechnology Institute, Federal University of Alagoas, Cidade Universitária, BR 101, Km 14, Norte Tabuleiro dos Martins, 57072-970 Maceió, Alagoas, Brazil; E-Mail: aegs@qui.ufal.br; 5 Chemistry Department, Federal University of Minas Gerais, Av. Pres. Antônio Carlos, 6627, Campus Pampulha, 31270-901, Belo Horizonte, MG, Brazil; E-Mail: lcab@ufmg.br; 6 Department of Fundamental Chemistry, Chemistry Institute, University of São Paulo, 05508-000 São Paulo-SP, Brazil; E-Mail: giovanacf@gmail.com; 7 Department of Plant and Animal Science, State University of Southeast of Bahia, Estrada do Bem Querer, Km 04, s/n, 45.083-900 Vitória da Conquista, Bahia, Brazil; E-Mail: mfigue2@uesb.edu.br; 8 Department of Exact and Technological Sciences, State University of Santa Cruz, Rodovia Ilhéus/Itabuna, Km 16, Salobrinho, 45662-900 Ilhéus, Bahia, Brazil; E-Mail: faustino@uesc.br

**Keywords:** antibacterial activity, *Prosopis juliflora* pods, juliprosopine (juliflorine), prosoflorine, juliprosine, *in vitro* ruminal digestion, feed additives

## Abstract

The purpose of this study was to assess the *in vitro* antimicrobial activity of alkaloid-enriched extracts from *Prosopis juliflora* (Fabaceae) pods in order to evaluate them as feed additives for ruminants. As only the basic chloroformic extract (BCE), whose main constituents were juliprosopine (juliflorine), prosoflorine and juliprosine, showed Gram-positive antibacterial activity against *Micrococcus luteus* (MIC = 25 μg/mL), *Staphylococcus aureus* (MIC = 50 μg/mL) and *Streptococcus mutans* (MIC = 50 μg/mL), its influence on ruminal digestion was evaluated using a semi-automated *in vitro* gas production technique, with monensin as the positive control. Results showed that BCE has decreased gas production as efficiently as monensin after 36 h of fermentation, revealing its positive influence on gas production during ruminal digestion. Since *P. juliflora* is a very affordable plant, this study points out this alkaloid enriched extract from the pods of *Prosopis juliflora* as a potential feed additive to decrease gas production during ruminal digestion.

## 1. Introduction

Since the last century, increasing atmospheric concentrations of methane have been reported [1,2], which has aroused worldwide interest in reducing emissions of enteric greenhouse gases into the atmosphere [3]. Approximately 15% of global CH_4_ emissions are produced by domestic ruminants during their digestive fermentation [4] and released into the environment by eructation. Ruminants typically lose 2%–12% of gross energy intake during this process [1]: this is a further reason why feed additives, such as ionophores, are used to reduce enteric CH_4_ emissions and improve feed conversion efficiency by diminishing acetic and butyric acid production in favor of propionic acid, which is more energetically efficient [5].

Considering the risks of antibiotic resistance in humans [6] and the occurrence of residues in foods of animal origin [7,8], the European Union has banned antibiotic use in livestock as feed additives, discontinuing a nearly 50-year period of antibiotic use for non-therapeutic purposes [8]. Thus, there is a real and growing demand for new feed additives to replace these compounds. In this connection, the scientific community initiated efforts to exploit natural products as feed additives, since many natural compounds and plant extracts afford some of the benefits of antibiotics [8,9].

*Prosopis* (Fabaceae) is a genus of about 45 species of spiny leguminous trees and shrubs found in subtropical and tropical regions of the Americas, Africa and southwest Asia. *Prosopis juliflora* (Sw.) D.C., commonly known as “algaroba” or “mesquite”, is a small perennial tree native to arid and semi-arid regions of Mexico, South America and the Caribbean and has established itself as a weed, notably in Asia and Australia. *P. juliflora* is found in Kuwait as a roadside tree and has an exceptional capacity to survive in arid-desert environments, at high temperatures, since its roots may grow at a depth of 53 m [10].

*P. juliflora* pods are characterized by elevated sugar content, about 300 g/kg of dry matter (DM). With 120 g/kg of crude protein on a DM basis, they have been used as human food and livestock feed for thousands of years in arid and semi-arid regions [11]. Manually ground, the pods make a flour used for diverse culinary purposes. Once concentrated, the aqueous extract obtained from these beans becomes dark and dense and can be used in beverages and jellies. Roasted and ground, the beans can be used to make a coffee-like beverage [11,12].

The pods of *P. juliflora* contain anti-nutritional factors, such as toxins and polyphenolics, which limit their utilization as an animal feed [13,14]. Intoxication by algaroba, popularly known as “twisted face”, has been found in cattle in the United States, Peru and Brazil, as well as in goats in Peru [15]. Clinical signs, including mandibular tremors, intensive salivation, difficulty of swallowing and torsion of the head, among other symptoms, which are induced from interruption of cranial nerves function, are more evident during rumination [16]. The long-term ingestion of algaroba in cattle diets can result in death. In order to avoid intoxication, cattle are fed rations containing no more than 40 g of dry algaroba beans/100 g DM, especially if the feeding period exceeds 60 days [17].

Numerous chemical constituents in the classes of flavonoids, piperidinic alkaloids and elagic acid glycosides have been isolated from *P. juliflora*, notably from its roots, stems and leaf [18–27]. Extracts (Table 1) and piperidinic alkaloids (Table 2) from *P. juliflora* leaves were shown to exhibit antimicrobial activity against several Gram-positive bacteria and fungi [24,25,28–32]. As far as the authors are aware, only four phytochemical studies on algaroba’s pods are available [15,16,33,34], and just one reports an antimicrobial evaluation of their extract [34]. A preliminary study by Batatinha [33] using an artificial rumen (RUSITEC) revealed that the alkaloidal fraction from the *P. juliflora* pods increased the amount of thiamine, thiamine diphosphate, thiamine monophosphate, protein, propionic and valeric acids, whereas it decreased the production of acetic and *i*-valeric acids after 23 days of fermentation, at the highest alkaloid concentration.

This study describes the antimicrobial activity against Gram-positive bacteria of the alkaloid-enriched extract obtained from *P. juliflora* pods by chloroform extraction. Since Archaea include the main agents responsible for CH_4_ and CO_2_ production during ruminal digestion [35], this study has also evaluated the influence of this extract on ruminal digestion by determining gas production, true degradability and microbial mass production using a semi-automated *in vitro* gas production technique [36], in order to compare it to monensin, an ionophore, which selectively inhibits rumen microbial growth.

## 2. Results and Discussion

The Dragendorff’s reagent, whose composition generally consists of an acidic solution of the iodide complex of bismuth (III), has been a practical chemical tool commonly used for thin layer chromatography (TLC) detection and identification of alkaloids since 1867 [37,38]. On TLC, Dragendorff-positive substances usually appear as orange spots on yellowish to brownish colored background [39].

**Figure 1:**
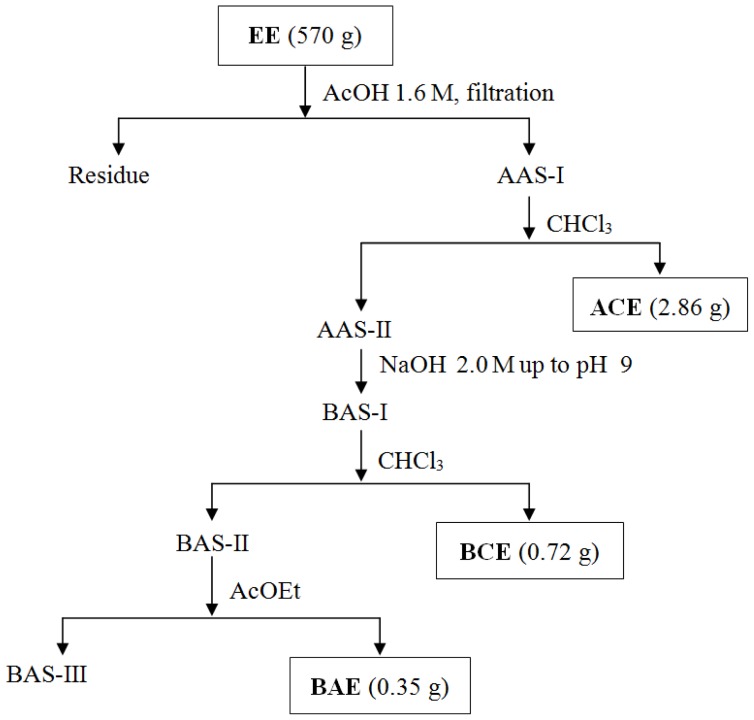
Acid-base fractionation of the ethanolic extract directed to isolation of alkaloids. Adapted from [40].

The crude ethanolic extract (EE) of *P. juliflora* pods exhibited Dragendorff-positive spots when subjected to TLC analysis, and, thus EE was submitted to the acid-base treatment in order to obtain extracts enriched with alkaloids. Firstly, EE was suspended in a 1.6 M AcOH solution, and then the resulting acid aqueous phase was filtered and extracted with chloroform and ethyl acetate at different pH values, leading to the corresponding acid chloroformic (ACE), basic chloroformic (BCE) and basic ethyl acetate (BAE) extracts (Figure 1). Only BCE and BAE were shown to be reactive on TLC under Dragendorff reaction.

Considering that previous works have reported antimicrobial activities of extracts and alkaloids obtained from the leaves of *P. juliflora* (Tables 1 and 2), it was proposed to evaluate the antimicrobial activity of the basic extracts BCE and BAE, both of them obtained from the acid-base fractionation of EE (Figure 1). These alkaloidal-enriched extracts were then assayed against the four Gram-positive bacteria, *Bacillus subtilis*, *Staphylococcus aureus, Streptococcus mutans* and *Micrococcus luteus*, the three Gram-negative bacteria, *Escherichia coli*, *Pseudomonas aeruginosa* and *Salmonella choleaesuis*, and against the three fungi, *Aspergillus niger*, *Cladosporium cladosporioides* and *Candida albicans*.

Results (Table 3) revealed that the basic ethyl acetate extract (BAE) did not present any antimicrobial activity against all microorganisms assayed (MIC > 100 μg/mL). The absence of antimicrobial activity for BAE may be explained, at least in part, by the low concentration of alkaloids in this extract. On the other hand, the basic chloroformic extract (BCE) was shown to be active against *M. luteus* (MIC = 25 μg/mL), *S. aureus* (MIC = 50 μg/mL) and *S. mutans* (MIC = 50 μg/mL), *M. luteus* being the microorganism more susceptible to this extract.

**Figure 2:**
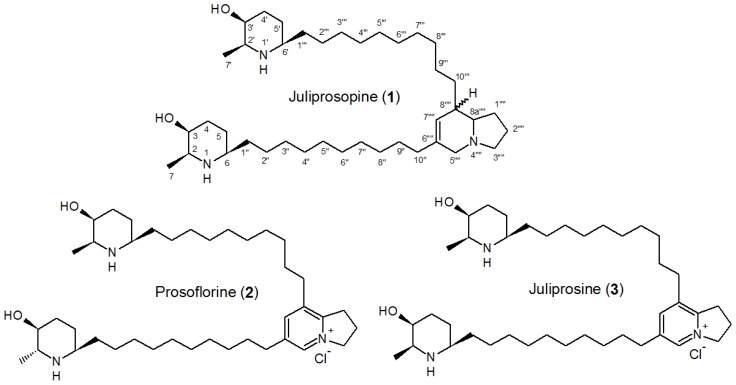
Chemical structures of juliprosopine (**1**), prosoflorine (**2**) and juliprosine (**3**).

**Figure 3:**
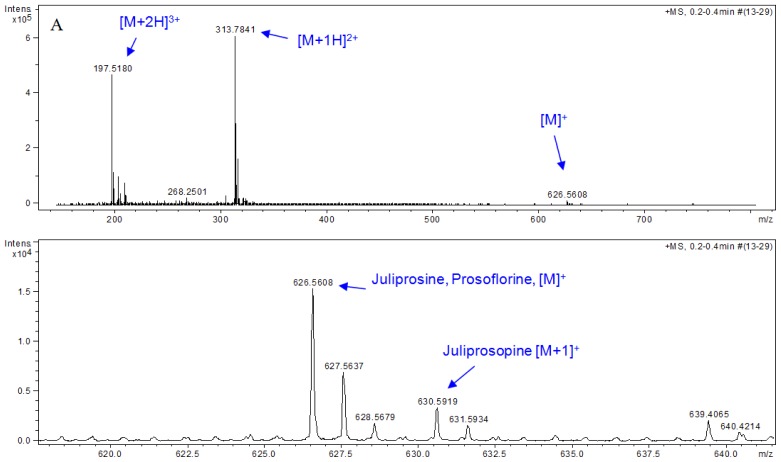

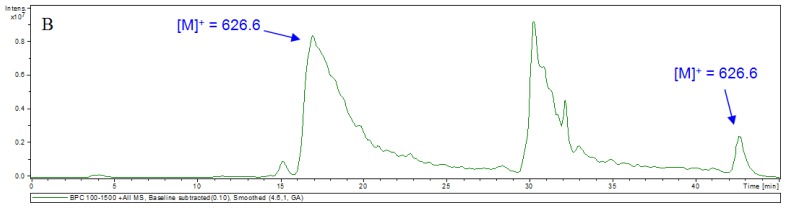
HRESIMS and HPLC-MS analyses of BCE. (**A**) Direct injection of BCE onto HRESIMS apparatus; (**B**) HPLC-MS chromatogram obtained for BCE. Chromatographic conditions: see experimental section.

A preliminary analysis of BCE and BAE was carried out by NMR. The ^1^H NMR spectrum of BCE (CDCl_3_, 200 MHz) showed signals at δ 9.04 (*s*, H-5″″), δ 7.76 (*s*, H-7″″), δ 5.28 (*br s*, H-3″″), δ 3.51 (*br s*, H-3 and 3′), δ 3.32 (*m*, H-1″″, H-2,2′), δ 1.11 (*m*, H-2″ to H-8″, H-2‴ to H-8‴), δ 1.11 and δ 1.07 (each *d*, *J* = 6.4 Hz, H-7 and H-7′), which were attributed to prosoflorine (**1**) [40]. The relative intensity of all of these signals allowed us to consider this alkaloid as the major constituent of BCE. ^13^C NMR spectrum (50 MHz, CDCl_3_) of this extract also showed characteristic signals of juliprosopine (**2**), also known as juliflorine [15,24] and juliprosine (**3**) [15,26,40] (Table 4), indicating these alkaloids (Figure 2) as the minor alkaloids of BCE. Although juliprosopine and juliprosine have already been reported previously as chemical constituents of the pods of *P. juliflora*[15,34], this study reports for the first time the occurrence of prosoflorine in this part of this plant. In order to confirm this chemical constitution, a sample of BCE was directly injected onto a HPLC-MS apparatus, and mass data were collected. Peaks at *m/z* 626.5608 (calculated (calcd.) for C_40_H_72_N_3_O_2_, M^+^ = 626.5624) and 630.5919 (calcd. for C_40_H_76_N_3_O_2_, [M+H]^+^ = 630.5937) were compatible with prosoflorine, juliprosine and juliprosopine, respectively (Figure 3), corroborating the conclusion that these compounds are the main piperidinic alkaloids of BCE.

The ^1^H NMR spectrum of BAE (CD_3_OD, 400 MHz), on the other hand, did not indicate any profile characteristic of piperidinic alkaloids, suggesting that such alkaloids are present in BAE as very minor constituents.

Since the alkaloid-enriched extract BCE obtained from the pods of *P. juliflora* was shown to display antimicrobial properties against Gram-positive bacteria (Table 3), whose cell wall structure may present certain similarities to Archaea, the main one responsible for the undesired production of CH_4_ during ruminal digestion [35], and considering that algaroba’s alkaloidal fractions have already altered ruminal metabolites production during a preliminary *in vitro* study conducted by Batatinha [33], the next step of this work was to evaluate the influence of BCE on ruminal digestion by analyzing gas production, true degradability of DM and microbial mass production using the semi-automated *in vitro* gas production technique [36], in order to compare its performances with those of monensin, a typical ionophore of polyether-type antibiotic that selectively inhibits the rumen microbial growth.

**Figure 4:**
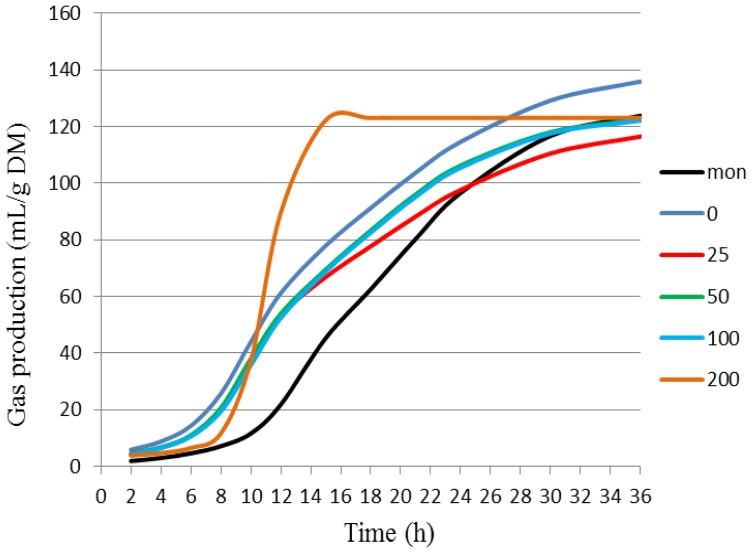
Cumulative gas production (mL/g DM) during ruminal fermentation in samples containing increasing concentrations of BCE (*i.e*., 0, 25, 50, 100 and 200 mg/L) or monensin (Mon, 5 μM), adjusted by the dual-pool model [41].

Figure 4 depicts cumulative gas production as a function of increasing BCE concentrations (*i.e.*, 0, 25, 50, 100 and 200 mg/L) or monensin (5 μM) at 2, 4, 6, 8, 10, 12, 15, 18, 21, 24, 30 and 36 h of incubation, according to the dual-pool model [41]. Table 5 presents kinetics parameters of gas production for each treatment after 36 h incubation, while Table 6 shows average values of cumulative gas production obtained at 6, 12, 18, 24 and 36 h of incubation. As it can be noted in Figure 4 and Table 6, both monensin and BCE treatments affected significantly the cumulative gas production in comparison to the control. BCE displayed a cumulative gas production closer to the control concerning the rapid degradation fraction (Table 5). In addition, monensin showed similar rates of degradation of soluble fractions (rapid degradation), but reduced their gas production. The longest time for the colonization of the fibrous fraction (lag time) was observed when monensin was used, which showed a higher degradation rate of this fraction (Table 5). There was interaction between treatments and time (*p* < 0.0001). The use of both monensin and BCE at different concentrations was effective in reducing the gas production after 36 h of incubation (Figure 4, Table 6).

Supplementary material (Tables S1 and S2) contains cumulative gas production data estimated by the dual-pool model for each treatment along 36 h of incubation, respectively, for fractions of fast and slow degradation. BCE 200 mg/L was effective in reducing the gas production until 12 h of incubation when compared to the control, which showed higher cumulative gas production from the degradation of soluble fraction (Table S1). After 18 h of incubation, both monensin and BCE had a similar performance to the control, indicating that this period of time was enough to promote degradation of soluble fractions (Table S1). Table S2 presents the estimative of cumulative gas production from degradation of fibrous fraction and shows that BCE 200 mg/L differed from both the control and monensin until 8 h of incubation. From 10 to 15 h, this treatment caused a similar effect to that observed for monensin. After 24 h of incubation, the dual-pool model estimated values for accumulated volume of gas that were similar to the control, monensin and levels of BCE (Table S2).

Assuming that there was no a general unspecific inhibition of rumen fermentation, the observed reduction in gas production by BCE can be understood as a result of a change in the ratio of volatile fatty acids (VFA, acetate/propionate) into an increasing molar ratio of propionate during fermentation, and this change may be attributed to the partial inhibition of cellulose-fermenting bacteria, which produce acetate, and of formate and H_2_ producers, which are the main precursors for CH_4_ biosynthesis [42]. Thus, this effect promotes simultaneously a higher sequestration of carbon in the culture medium and a lower production of CO_2_ and CH_4,_ improving the food conversion efficiency and ensuring a greater supply of glucose to ruminants during critical phases of their metabolism, maintaining a sparing effect of amino acids degraded to produce glucose. As CO_2_ and CH_4_ are the gases that contribute most to the increase in pressure in the hatchery environment, the presumed reduction of both can easily explain the reduction in the estimated volume of the gases. Similar results were described by Junior and co-workers [43], who evaluated the use of propolis extract on the gas production *in vitro* using different substrates.

According to the literature, piperidinic alkaloids of *P. juliflora*, such as julifloricine, juliprosinene and juliflorine (Table 2), affect the growth of Gram-positive bacteria, a group that include the fibrolytic bacteria, and is closely related to methanogenic Archaea [44], which is the greatest one responsible for the CO_2_ and CH_4_ production, respectively. The mechanism of action of piperidinic alkaloids on Gram-positive bacteria is due to its high cytotoxicity generated by blocking calcium channels in the cell membrane, mainly on account of the amphoteric characteristics of these alkaloids, which allow them to interact more efficiently with the cell membrane and inhibit its channels [45].

Both monensin and BCE have decreased the gas production over 36 h of incubation when compared to the control (Figure 4, Table 5). Hence, one may infer that the alkaloids extracted from the pods of *P. juliflora* might have mitigated the CH_4_ production by destabilizing selectively the syntrophic relationships between methanogenic microbial community and fibrolytic bacteria. The presence of methanogens in the anaerobic medium removes H_2_ to produce CH_4_, decreasing the partial pressure of H_2_ in this medium and, consequently, imposing a NAD (nicotinamide adenine dinucleotide) regeneration through other pathways, which may vary depending on the involved fibrolytic species [46,47]. Thus, species involved in syntrophic relationships grow better in the presence of methanogens, and therefore, there is increased microbial mass.

Interestingly, monensin did not provoke the expected decreasing effects on gas volume from degradation slow fraction over 36 hour’s incubation (Table 6). This ionophore is reported to inhibit ruminal production of both CH_4_ and CO_2_[5,42], but in some specific conditions and substrates, this inhibition seems to demand longer time to be evident, notably after 48 h of incubation [48]. Considering that ionophore antibiotics are not inhibitory to methanogenic bacteria, the lower gas production is believed to be due to the lower production of the precursors H_2_ and formate [49,50], probably by inhibiting fibrolytic bacteria, which produce hydrogen, and by favoring the syntrophic relationship between Gram-negative bacteria, such as *Fibrobacter succinogenes* and *Selenomonas ruminantium*. These bacteria, among others, oxidize H_2_ by using fumarate as the final electron acceptor, suggesting that they compete with methanogens for H_2_, which is the major substrate for methanogenesis in the rumen [51]. In addition, formate may be used as another electron donor for fumarate reduction [51].

The results concerning the true degradability of DM (TDDM) of the control in comparison to those of samples containing monensin (5 μM) or increasing concentrations of BCE (mg/L) after 18 and 36 h of incubation are presented in Table 7. The lowest TDDM value was observed for samples containing BCE 200 mg/L after 18 h of incubation. It was also verified that monensin and BCE 200 mg/L have significantly reduced the TDDM in comparison to the control (BCE 0) and to the other BCE treatments (25, 50 and 100 mg/L) at 18 h of incubation; at 36 h, in turn, only monensin presented lower TDDM, as compared to the other treatments (Table 7).

BCE at 200 mg/L and monensin were shown to significantly reduce TDDM in comparison to the other treatments after 18 h (Table 7); this fact may be explained by the inhibition of fibrolytic microorganisms, resulting in lower degradation of fibrous components.

As regards dry microbial mass production (DMMP), Table 8 shows that BCE 50, BCE 100 and BCE 200 mg/L have significantly produced far less dry microbial mass than BCE 0, BCE 25 and monensin at the time of 18 h, whereas only monensin has significantly differed from the control at 36 h. Furthermore, it should be noted that only monensin has significantly increased the parameter in question at 36 h of incubation, as compared to the control and other treatments (Table 8).

Despite BCE having been as efficient as monensin in provoking lower total gas production during the 36 h incubation (Tables 5, S1 and S2), monensin has caused the lowest degradation and the highest microbial yield (Tables 7 and 8). Thus, BCE was shown to promote the most selective reduction on gas production, since substrate degradation was less affected by this extract than by monensin. Anyway, further studies should be addressed in order to confirm that rumen fermentation has not been inhibited by BCE. Moreover, further investigations will elucidate, on the basis of VFA profile information, whether any inhibition caused by this extract is indeed selective or not. It is very interesting to ruminants that fermentation promotes the maximum microbial synthesis and high short-chain fatty acids production, especially propionate, with lower gas and heat in fermentation.

Thus, taking into account that *P. juliflora* is a very affordable plant, the present study points out BCE as a promising starting material for further studies focusing on the development of novel feed additives to decrease CH_4_ and CO_2_ production during ruminal digestion.

## 3. Experimental Section

### 3.1. General Procedures

Proton nuclear magnetic resonance (^1^H NMR) spectra were recorded at 400 MHz using deuterochloroform (BCE) or deuteromethanol (BAE) as solvents and tetramethylsilane (TMS) as the internal reference, on a Bruker Advance III (200 MHz) and a Bruker ARX400 (400 MHz) apparatus (Bruker AXS, Inc., Madison, WI, USA). Chemical shift values were expressed in ppm and coupling constants (*J*) in Hz. Thin layer chromatographies (TLC) were on 0.25 mm thick silica gel Merck 60 F254 (Merck & Co., Inc., Whitehouse Station, NJ, USA). Solvents and reagents were purified by standard procedures as necessary.

### 3.2. Preparation of the Dragendorff’s Reagent

Preparation of the Dragendorff’s reagent was adapted from literature [52]. Basic bismuth nitrate (1 g) was dissolved in the pre-mixed solvents (10 mL of concentrated hydrochloric acid + 40 mL distilled water). Next, 5 g of potassium iodide was fully dissolved in this solution, which was then completed to 100 mL with distilled water and subjected to TLC detection directly.

### 3.3. Plant Material

Pods of *Prosopis juliflora* were collected manually in November of 2005 in Brumado, Bahia, Brazil. A voucher specimen was housed at the Herbarium of the Universidade Estadual de Santa Cruz (UESC), in Itabuna, Bahia, Brazil, under the code RG-14435. The plant material was dried at 25 to 30 °C in a well-ventilated area and then pulverized in a mill to create a yellowish dry powder (2.6 kg).

### 3.4. Obtaining Extracts

All powdered plant material was initially extracted by percolation with hexane (10 L), giving, after concentration in a rotary evaporator (40 °C), the hexanic extract (HE, 13.0 g). Next, the defatted plant material was extracted with ethanol (10 L) by percolation, and this solution was concentrated under reduced pressure to yield the crude ethanolic extract (EE, 870 g).

This extract received acid-base treatment according to the Ott-Longoni and co-workers’ methodology [40] to isolate alkaloids, whose adapted procedure is presented in Figure 1. Part of the EE (570 g) was made soluble in aqueous 1.6 M acetic acid (AcOH, 500 mL), and the resulting solution was filtered to yield the acid aqueous solution I (AAS-I). This was extracted with CHCl_3_ (2 × 300 mL), and the remaining aqueous solution was renamed as the acid aqueous solution II (AAS-II). This CHCl_3_ extract was washed with NaHCO_3_ 0.6 M (2 × 300 mL) and brine (2 × 300 mL) and then dried with Na_2_SO_4_ and concentrated under reduced pressure, thereby creating the acid chloroformic extract (ACE, 2.86 g). Next, the AAS-II was neutralized with NaOH 2.0 M up to pH 9.0, leading to the basic aqueous solution I (BAS-I), which was, in turn, extracted immediately with CHCl_3_, and the resulting organic layer was washed with brine, dried with Na_2_SO_4_ and concentrated under reduced pressure to yield the basic chloroformic extract (BCE, 0.72 g). The BAS-I was, at this point, termed the basic aqueous solution II (BAS-II) and extracted with AcOEt. The resulting organic extract was washed with brine, dried with Na_2_SO_4_ and concentrated under reduced pressure to give the basic aqueous solution III (BAS-III) and the basic acetate extract (BAE, 0.35 g) residue.

### 3.5. HPLC-MS Analyses

Analytical HPLC was performed in a Shimadzu chromatograph model SLC-10A (Tokyo, Japan), solvent pumps (LC-10AD) and a Phenomenex Gemini C-18 column (250 × 4.6 mm; 5μm). The solvents, methanol (B) and 0.1% formic acid (A), were used as the mobile phase in the following gradient elution: 0–5 min, 30% B; 5–30 min, 30%–100% B; 30–38 min, 100% B; 38–40 min, 100%–30% B; 40–45 min, 30% B. Injection volume: 20 μL of a MeOH solution (1 mg•mL^−1^), room temperature. The LRESIMS/MS was measured on a Bruker Esquire 3000 plus spectrometer (Bremen, Germany) with an Ion trap analyzer and electrospray ionization. HRESIMS was measured on a Bruker micrOTOF spectrometer (Bremen, Germany), with a time-of-flight analyzer and electrospray ionization.

### 3.6. *In Vitro* Antimicrobial Assay

For tests of antimicrobial activity, extracts were assayed against the Gram-positive bacteria, *Bacillus subtilis* (ATCC 6633), *Staphylococcus aureus* (ATCC 6538), *Streptococcus mutans* (ATCC 25175) and *Micrococcus luteus* (ATCC 10240), the Gram-negative bacteria, *Escherichia coli* (ATCC 94863), *Pseudomonas aeruginosa* (ATCC 9027) and *Salmonella choleaesuis* (ATCC 14028), and the fungi, *Aspergillus niger* (ATCC 16404), *Cladosporium cladosporioides* (IMI 178517) and *Candida albicans* (ATCC 18804).

Minimum inhibitory concentration (MIC) values of each Dragendorff-positive extract were determined by the broth microdilution method using 96 well microplates, as described previously [53,54]. The bacteria were cultured in nutrient broth (Oxoid) for 24 h at 37 °C. The filamentous fungi were cultured in malt extract (Acumedia) and the leveduriform fungus *C. albicans* in yeast malt extract broth (Acumedia), for 72 h at 26 °C. Each extract was diluted in a stock solution prepared in water-dimethyl sulfoxide (80:20 v/v) and assayed at the final concentrations of 100, 50, 25, 12.5, 6.3, 3.1, 1.6 and 0.78 μg/mL. The positive controls were chloramphenicol for bacteria and cyclopirox olamine (Loprox) for fungi. The initial microorganisms’ inocula were adjusted to the turbidity of 0.5 McFarland, and the final concentration was 0.9 × 10^6^ cells/well. Samples were assayed in triplicate. The MIC value was defined as the minimum concentration in which a given extract inhibited visible growth.

### 3.7. Evaluation of Effects on Digestion Parameters by *in Vitro* Gas Production

This evaluation was according to the Mauricio and co-workers’ methodology [36]. Rumen fluid was collected manually from the ventral sac of the rumen of a fistulated dry cow after a 12 h fasting. Rumen contents were strained using a double layer of muslin cloth directly to a pre-heated vacuum flask (39 °C), and the resulting rumen fluid was transported immediately to the laboratory, where it remained for 30 min to decant heavier particles. The rumen fluid donor cows were under pasture conditions (*Brachiaria decumbens*) and supplemented with 2 kg wheat bran fed once a day. A buffer solution (200 mL) containing NH_4_HCO_3_ (4.5 g/L) and NaHCO_3_ (39.4 g/L) was mixed with distilled water (500 mL), micromineral solution (200 mL; CaCl_2_•2H_2_O, 148.5 g/L; MnCl_2_•4H_2_O, 112.5 g/L; CoCl_2_•6H_2_O, 11.3 g/L; FeCL_3_•6H_2_O, 90.0 g/L), macromineral solution (0.1 mL; Na_2_HPO_4_•12H_2_O, 10.6 g/L; KH_2_PO_4_, 7.0 g/L; MgSO_4_•7H_2_O, 0.7 g/L), reducing solution (60 mL; cysteine•HCl, 7.0 g/L; Na_2_S•9H_2_O, 7.0 g/L; NaOH, 1.8 g/mL) and resazurin solution (1.0 mL; 0.01 g/L) to prepare the predigestion solution. In order that the *in vitro* rumen fermentation might take place, 10 mL of rumen fluid and 90 mL of predigestion solution were added to each 160 mL flask, and then the digestion medium was obtained. The digestion medium was prepared in triplicate, being each a replicate from a rumen fluid of a different cow.

The alkaloid enriched extract (BCE, 200 mg) was dissolved in dimethyl sulfoxide (10 mL), and aliquots of this solution were transferred to flasks containing the digestion medium (initially with 90 mL, then completed to 100 mL), in order to create different concentrations of extract (*i.e.*, 0, 25, 50, 100 and 200 mg/L) with use of no more than 1 mL of dimethyl sulfoxide/100 mL of solution. Each treatment was assayed in triplicate, using three different replicates of digestion media. prepared as described above. Monensin was the positive control (5 μM), and wheat bran (1 g) was used as the substrate during incubation. Substrates were incubated with this buffered rumen fluid and the growth medium prepared according to the Manual for gas production technique, Institute of Grassland and Environmental Research (IGER), with adaptations from the Theodorou and co-workers’ procedure [55], in sealed fermentation flasks of 160 mL, which were previously saturated with CO_2_. A pressure transducer with a digital output display (T443A, Bailey and Mackey, Birmingham, England) was interfaced with a computer using a K485 converter (Shenzhen ATC Technology Co. Ltd., Shenzhen, China). The headspace gas pressure was measured by inserting a hypodermic needle, attached to the hand-held pressure transducer, through the butyl-rubber stopper of the fermentation flask, allowing accumulated head-space gas pressure values to be directly entered into a spreadsheet. At the beginning of fermentation, zero gas production was assumed. From each value of gas pressure obtained at a given time, gas production was calculated in the period using a previous quadratic equation, which correlates gas production and gas pressure adjusted for the local altitude where the experiment was held. Pressure measurements were made twice at 2, 4, 6, 8, 10, 12, 15, 18, 21, 24, 30 and 36 h. After being measured, gas was released from each fermentation flask and data were used to generate gas volume estimates through the quadratic function:

$$V(\text{mL})=-0.02+4.30p+0.07p^{2},\;\text{where}:\text{p}=\text{pressure }(\text{psi}).$$

Then, flasks were swirled and returned to the incubator. Fermentation was terminated by decreasing the temperature of the flasks to 4 °C, and substrate degradability was estimated as the difference between the amount of sample placed in each bottle to ferment and the amount of residue recovered by filtering the residues with Gooch (sintered glass) crucibles (porosity 1, Vidrotec^®^, Vidrotech Equipamentos para Laboratorios, Blumenau, Brazil) under vacuum.

To determine DM disappearance, bottles were removed at pre-set times (18 and 36 h) and the resulting residue directly filtered and oven dried at 105 °C for 16 h to evaluate the apparent degradability of DM (ADDM). For determination of true degradability of DM (TDDM), the incubation residues were digested in neutral detergent to remove microbial mass [56]. Dry microbial mass production (DMMP) was estimated by the difference between ADDM and TDDM and expressed in mg/100 mg of degradable DM.

### 3.8. Statistical Analysis

For the analysis of cumulative gas production, the dual-pool model approach [41] was used to estimate the ruminal fermentation dynamics, whose equation used was:

$$V=VFF/[1+{\text{e}}^{2+4KdFF\cdot (L-T)}]+VSF/[1+{\text{e}}^{2+4KdSF\cdot (L-T)}]$$

where: *V* = accumulated gas volume along the time; *VFF* = gas volume from the fast fraction; *VSF* = gas volume from the slow fraction; *KdFF* and *KdSF* = Degradation rates of fast and slow fractions (h^−1^), respectively; *L* = lag time (h); and *T* = 2, 4, 6, 8, 10, 12, 15, 18, 21, 24, 30 and 36 h.

Data analysis was by the MIXED procedure of SAS [57], in repeated measure designs (times). The effects of treatments and times were decomposed into linear and quadratic polynomial contrasts. In addition, the interaction of treatments over time was examined using the contrasts. The lowest setting of the Akaike information criterion (AIC) was obtained using the variance component (VC):

$$Y_{ijk}=\mu +Tr_{i}+\delta_{j(i)}+Tk+TrxT_{ik}+{ɛ}_{ijk}$$

where: *Y_ijk_* is the response at time *k* on animal (rumen fluid donor) *j* in treatment additive *i; μ* is the overall mean; *Ti* is the fixed effect of additive *i* (*i =* control, dose of BCE or monensin); *Tr* = BCE (0, 25, 50, 100, 200 mg/L) and monensin (5 μM); *T* = time (2, 4, 6, 8, 10, 12, 15, 18, 21, 24, 30 and 36 h) for cumulative gas production and *T* = time (18 and 36 h) for TDDM and DMMP; *δ_j(i)_* is the random effect of inoculum *j* within treatment *i* (which was the term used as experimental error to test the effect of additive i); *Tk* is the effect of incubation time *k; TrxT_ik_* is the interaction effect of additive *i* with time *k*; and *ɛ_ijk_* is the residual error (random error at time *k* on animal *j* in additive *i*).

The contrasts of the interaction were employed to compare the effect of BCE levels and monensin and interactions over time on cumulative gas production, TDDM and DMMP.

## 4. Conclusions

Prosoflorine is described for the first time as a chemical constituent of the pods of *P. juliflora*. In addition, the results described in this study allow us to conclude that the antibacterial activity of BCE has a positive influence on gas production during ruminal digestion and its selectivity on ruminal microorganisms seems to be higher than that of monensin. Moreover, considering that *P. juliflora* is a very affordable plant occurring in arid and semi-arid regions of the world, the present study points out *P. juliflora* pods as a potential source for the development of an alternative feed additive that decreases the undesired production of CH_4_ and CO_2_ during ruminal digestion, reducing their emission into the atmosphere.

## Supplementary Information



## Figures and Tables

**Table 1:** Antimicrobial activities described for *Prosopis juliflora* extracts.

Part used	Extract (Method)	Susceptible Microorganisms	Concentration (MIC)	Reference
Leaves	Aqueous (maceration)	*Xanthomonas campestris*	50 g of leaves in 100 mL H_2_O	[29]

Leaves	Aqueous *	Fungi:		[25]
		*Allescheria boydii*	10 g/mL	
		*Aspergillus niger*	7.5 g/mL	
		*Aspergillus fumigatus*	10 g/mL	
		*Aspergillus flavus*	15 g/mL	
		*Candida albicans*	0.5 g/disc	
		*Candida tropicalis*	0.5 g/disc	

Leaves	Methanolic	Gram-positive Bacteria:		[30]
		*Staphylococcus aureus*	1 g/disc	
		*Bacillus subtilis*		
		*Sarcina lutea*		
		*Streptococcus pyogenes*		
		*Streptococcus faecalis*	10 g/disc	
		Gram-negative Bacteria:		
		*Escherichia coli*	1 g/disc	
		*Klebsiela pneumonia*		
		*Samonella typhi*		
		*Proteus ulgaris*		
		Fungi:		
		*Candida albicans*	30 g/disc	
		*Rhizopus nigricans*	30 g/disc	
		*Aspergillus flavus*	40 g/disc	
		*Aspergillus nidulans*	20 g/disc	

Leaves	Hydroalcoholic (maceration)	*Neisseria gonorrhoeae*	50 mg dried leaves/disc	[31]

Pods	Alkaloid Rich Fraction *	Gram-negative Bacteria:		[34]
		*Escherichia coli*	75 g/mL	
		*Klebsiella pneumonia*	75 g/mL	
		*Pseudomonas putida*	50 g/mL	

Pods	Basic Chloroformic *	Gram-positive Bacteria:		Present study
		*Micrococcus luteus*	25 g/mL	
		*Staphylococcus aureus*	50 g/mL	
		*Streptococcus mutans*	50 g/mL	

* Through acid-base fractionation for obtaining alkaloid-enriched extracts. MIC, minimum inhibitory concentration.

**Table 2:** Antimicrobial activities described for isolated *Prosopis juliflora* alkaloids.

Alkaloid	Part	Susceptible Microorganisms	Concentration	Reference
Julifloricine	Leaves	Gram-positive Bacteria:		[28]
		*Staphylococcus aureus*	1 g/mL	
		*Staphylococcus citrus*		
		*Staphylococcus epidermidis*		
		*Staphylococcus pyogenes*		
		*Sarcita lutea*		
		*Staphylococcus faecalis*	5 g/mL	
		*Staphylococcus pneumoniae*		
		*Staphylococcus lactis*		
		*Corynebacterium diphtheriae*		
		*Corynebacterium hofmanii*		
		*Bacillus subtilis*		
		Fungi:		
		*Candida albicans*	2.5 g/mL	
		*Candida tropicalis*	1.0 g/mL	

Juliprosinene	Leaves	Bacteria:		[24]
		*Escherichia coli*		
		*Klebsiella pneumoniae*		
		*Pseudomonas aeruginosa*	N.D. *	
		*Staphylococcus aureus*		
		*Shigella sonnei*		

Juliflorine (Juliprosopine)	Leaves	Gram-positive Bacteria:		[32]
		*Streptococcus pyogenes*	1 to 30 g/mL	
		*Staphylococcus aureus*		
		*Corynebacterium diphtheriae*		
		*Corynebacterium hofmanni*		
		*Bacillus subtilis*		
		*Streptococcus faecalis*		
		Fungus: *Candida sp*.	0.5 to 5 g/mL	
		Dermatophyte Fungi	2.5 g/mL	
		Protozoa:		
		*Entamoeba histolytica*	10 g/mL	

* N.D., not described.

**Table 3:** Minimum inhibitory concentration (MIC, μg/mL) for basic chloroformic (BCE) and basic ethyl acetate (BAE) extracts of *P. juliflora* pods against some microorganisms.

Microorganisms	BCE	BAE	Chloramphenicol a	Loprox b
*M. luteus*	25	>100	0.8	-
*S. aureus*	50	>100	6.3	-
*S. mutans*	50	>100	6.3	-
*B. subtilis*	>100	>100	6.3	-
*E. coli*	>100	>100	3.1	-
*S. choleaesuis*	>100	>100	6.3	-
*P. aeruginosa*	>100	>100	100	-
*A. Niger*	>100	>100	-	12.5
*C. cladosporioides*	>100	>100	-	6.3
*C. albicans*	>100	>100	-	6.3

a Positive control for bacteria;

b Ciclopirox olamine, positive control for fungi.

**Table 4:** Comparison of ^13^C NMR data (δ in ppm, 50 MHz, CDCl_3_) obtained for BCE with those reported in the literature for juliprosopine (**1**), prosoflorine (**2**) and juliprosine (**3**).

Carbon	Literature [24,26,40]			BCE		
	
	1	2	3	1	2	3
1″″	33.2	32.4	32.4	33.2	32.3	32.3
2″″	21.5	21.3	21.4	21.4	21.3	21.4
3″″	54.5	59.8	59.8	54.5	60.5	59.8
5″″	55.3	138.9	139.0	55.2	138.7	138.9
6″″	136.3	139.1	139.0	136.0	138.9	138.9
7″″	123.8	144.0	144.0	123.9	143.9	143.9
8″″	42.6	141.9	141.8	42.5	141.7	141.7
8a″″	65.5	154.2	154.0	65.5	154.1	154.1
2,2′	57.2	57.3	57.2	57.1	57.1	57.1
3,3′	67.8	67.6, 77.3	67.2	67.7	67.7, 77.4	67.3
4,4′	32.2	32.0, 31.7	31.8	32.3	31.9, 31.7	31.9
5,5′	26.2	25.7	25.6	26.6	25.6	25.5
6,6′	55.7	55.9	55.8	55.8	55.8	55.8
1″,1‴	37.1	36.2	36.1, 36.0	36.6	36.1	36.1, 35.9
2″,2‴	25.8	25.6	25.1, 25.0	25.8	25.6	25.1, 24.9
3″,8″	30.0–29.4	31.9–28.9	32.0, 30.8	30.0–28.8	30.0–28.8	31.9, 30.7
3‴–8‴	30.0–29.4	31.9–28.9	30.5–29.0	30.0–28.8	30.0–28.8	30.0–28.8
9″,9‴	26.6	N.A.*	N.A.*	26.6	N.A.*	N.A.*
10″	35.1	N.A.*	N.A.*	35.2	N.A.*	N.A.*
10‴	28.0	N.A.*	N.A.*	27.9	N.A.*	N.A.*
7,7′	18.7	18.1, 17.9	18.1, 18.0	18.4	18.0, 17.9	18.0, 17.9

* *N.A.*, not attributed.

**Table 5:** Kinetics parameters of gas production of samples containing increasing concentrations of BCE (mg/L) or monensin (Mon, 5 μM) after incubation of 36 h.

Treatment	Kinetic Parameters of Gas Production *				

	VFF 1	KdFF 2	L 3	VSF 4	KdSF 5
BCE 0	43.31 ± 0.69 ^a^	0.17 ± 0.01 ^a^	6.55 ± 0.22 ^c^	96.73 ± 3.46 ^a^	0.0434 ± 0.0005 ^c^
BCE 25	38.53 ± 2.58 ^a^	0.19 ± 0.01 ^a^	6.93 ± 0.27 ^b,c^	81.67 ± 13.58 ^a^	0.0453 ± 0.0005 ^b,c^
BCE 50	33.75 ± 1.44 ^a^	0.20 ± 0.01 ^a^	6.99 ± 0.25 ^b,c^	90.72 ± 2.38 ^a^	0.0497 ± 0.0005 ^b^
BCE 100	34.76 ± 1.21 ^a^	0.19 ± 0.01 ^a^	7.16 ± 0.10 ^b,c^	89.56 ± 2.99 ^a^	0.04933 ± 0.00009 ^b^
BCE 200	36.92 ± 3.81 ^a^	0.16 ± 0.01 ^a^	8.15 ± 0.54 ^b^	86.28 ± 7.64 ^a^	0.0445 ± 0.0019 ^c^
Mon	20.12 ± 0.62 ^b^	0.26 ± 0.07 ^a^	10.90 ± 0.32 ^a^	106.53 ± 1.30 ^a^	0.056 ± 0.001 ^a^

* Means ± standard deviation. Means followed by the same minuscule letter within each time period did not differ by contrasts (*p* > 0.05).

1 VFF, Gas volume for degradation of fast fractions (mL) (*p* = 0.0001);

2 KdFF, degradation rate of fast fractions (1/h) (*p* = 0.2762);

3 L, lag time (h) (*p* < 0.0001);

4 VSF, gas volume for degradation of slow fractions (mL) (*p* = 0.2128);

5 KdSF, degradation rate of slow fractions (1/h) (*p* < 0.0001).

**Table 6:** Cumulative gas production (mL/g of DM) of samples containing increasing concentrations of BCE (mg/L) or monensin (Mon, 5 μM) at 6, 12, 18, 24 and 36 h of incubation.

Treatment		Cumulative Gas Production (mL/g of DM) *				

		6 h	12 h	18 h	24 h	36 h
BCE	0	15.31 ± 1.26 ^a^	60.17 ± 1.52 ^a^	91.76 ± 1.75 ^a^	114.22 ± 2.63 ^a^	137.79 ± 2.80 ^a^
	25	11.00 ± 2.08 ^b^	53.23 ± 2.06 ^b^	81.21 ± 3.61 ^b^	100.44 ± 5.80 ^b,c^	120.89 ± 7.70 ^b^
	50	11.52 ± 0.67 ^b^	52.92 ± 1.07 ^b^	83.39 ± 0.67 ^b^	106.15 ± 1.07 ^b^	124.28 ± 1.31 ^b^
	100	10.81 ± 0.55 ^b^	51.66 ± 1.62 ^c^	82.89 ± 2.21 ^b^	105.64 ± 2.61 ^b^	123.48 ± 2.96 ^b^
	200	8.73 ± 1.39 ^c^	40.31 ± 3.10 ^d^	74.79 ± 2.75 ^b^	97.27 ± 3.75 ^c^	120.32 ± 4.00 ^b^
Mon		2.69 ± 0.36 ^d^	24.78 ± 1.88 ^e^	61.84 ± 1.98 ^c^	96.51 ± 1.53 ^c^	126.08 ± 1.19 ^b^

* Means ± standard deviation. Means followed by the same minuscule letter within each time period did not differ by contrasts (*p* > 0.05). Analysis of variance (Treatment: *p* < 0.0001, Time: *p* < 0.0001, Time × treatment: *p* < 0.0001).

**Table 7:** True dry matter degradability (TDDM, g/100 g DM) of samples containing increasing concentrations of BCE (mg/L) or monensin (Mon, 5μM), after incubation of 18 and 36 h.

Treatment	TDDM (g/100 g DM) *	

	18 h	36 h
BCE 0	69.25 ± 0.81 ^a^	72.72 ± 0.34 ^a^
BCE 25	70.61 ± 0.63 ^a^	71.77 ± 1.34 ^a^
BCE 50	69.54 ± 0.65 ^a^	73.07 ± 0.11 ^a^
BCE 100	69.15 ± 0.64 ^a^	72.38 ± 0.35 ^a^
BCE 200	65.73 ± 0.42 ^b^	71.41 ± 0.04 ^a^
Mon	57.04 ± 0.23 ^c^	62.21 ± 0.58 ^b^

* Means ± standard deviation. Means followed by the same minuscule letter within each time period did not differ by contrasts (*p* > 0.05). Analysis of variance (Time: *p* < 0.0001, Time × treatment: *p* < 0.0178).

**Table 8:** Dry microbial mass production (DMMP, mg/100mg degradable DM) of samples containing increasing concentrations of BCE (mg/L) or monensin (Mon, 5μM), after incubation for 18 and 36 h.

Treatment	DMMP (mg/100 mg Degradable DM) *	

	18 h	36 h
BCE 0	16.08 ± 0.72 ^a^	7.69 ± 0.62 ^a^
BCE 25	18.85 ± 1.38 ^a^	8.46 ± 0.33 ^a^
BCE 50	12.50 ± 0.05 ^b^	8.40 ± 0.24 ^a^
BCE 100	12.32 ± 0.70 ^b^	8.71 ± 1.95 ^a^
BCE 200	11.81 ± 0.10 ^b^	10.93 ± 2.06 ^a^
Mon	23.90 ± 0.64 ^a^	18.01 ± 1.64 ^b^

* Means ± standard deviation. Means followed by the same minuscule letter within each time period did not differ by contrasts (*p* > 0.05). Analysis of variance (Time: *p* < 0.0001, Time × treatment: *p* < 0.0273).

## Abbreviations

AAS: acid aqueous solution
ACE: acid chloroformic extract
ADDM: apparent degradability of DM
BAE: basic ethyl acetate extract
BAS: basic aqueous solution
BCE: basic chloroformic extract
DM: dry matter
DMMP: dry microbial mass production
EE: ethanolic extract
HRESIMS: high resolution electron spray ionization mass spectrometry
HPLC-MS: high-performance liquid chromatography mass spectrometer
^1^H NMR: proton nuclear magnetic resonance
MHz: megahertz
MIC: minimum inhibitory concentration
MMP: microbial mass production
RUSITEC: rumen simulation technique
SAEG: system of statistical and genetic analysis
TDDM: true degradability of DM
TLC: thin layer chromatography
TMS: tetramethylsilane
VFA: volatile fat acids
