# Supplementary material for: Antibacterial Activity of the Alkaloid-Enriched Extract from Prosopis juliflora Pods and Its Influence on in Vitro Ruminal Digestion
Source: Int J Mol Sci. 2013 Apr 17;14(4):8496–516. doi: 10.3390/ijms14048496 (PMC3645758; doi:10.3390/ijms14048496)
Supplement: Supplementary file 1 [file ijms-14-08496-s001.pdf]

Supplementary Information

Table S1. Cumulative gas production from degradation of fast fraction (mL/g of DM) estimated by dual-pool model from samples containing increasing concentrations of BCE (mg/L) or monensin (Mon, 5 μM) along 36 h of incubation.

Time (h)	Cumulative Gas Production from Degradation of Fast Fraction (mL/g of DM) *					
	Mon	0	25	50	100	200
2	0.001 ± 0.00 [a]	0.259 ± 0.08 [b]	0.111 ± 0.02 [b,c]	0.112 ± 0.02 [b,c]	0.109 ± 0.01 [b,c]	0.085 ± 0.09 [c]
4	0.006 ± 0.00 [a]	0.930 ± 0.21 [b]	0.489 ± 0.07 [b,c]	0.525 ± 0.07 [b,c]	0.478 ± 0.04 [b,c]	0.288 ± 0.29 [c]
6	0.03 ± 0.01 [a]	3.23 ± 0.49 [b]	2.11 ± 0.17 [b,c]	2.39 ± 0.14 [b,c]	2.04 ± 0.13 [b,c]	0.97 ± 0.97 [c]
8	0.14 ± 0.04 [a]	9.99 ± 0.75 [b]	8.03 ± 0.15 [b,c]	9.13 ± 0.05 [b]	7.58 ± 0.46 [b,c]	3.14 ± 0.28 [c]
10	1.01 ± 0.16 [a]	21.14 ± 0.59 [b]	22.95 ± 1.38 [b]	21.66 ± 0.25 [b]	19.26 ± 0.95 [b]	9.04 ± 0.41 [c]
12	6.48 ± 2.97 [a]	35.06 ± 0.61 [b]	32.70 ± 2.53 [b]	30.18 ± 0.73 [b]	29.37 ± 0.92 [b]	19.42 ± 0.57 [c]
15	15.65 ± 1.58 [a]	41.94 ± 0.64 [b]	37.83 ± 2.66 [a,b]	33.36 ± 1.32 [a,b]	34.07 ± 1.13 [a,b]	32.11 ± 2.22 [a,b]
18	19.51 ± 0.32 [a]	43.11 ± 0.68 [a]	38.45 ± 2.60 [a]	33.71 ± 1.42 [a]	34.68 ± 1.20 [a]	36.01 ± 3.44 [a]
21	20.06 ± 0.59 [a]	43.28 ± 0.68 [a]	38.52 ± 2.58 [a]	33.75 ± 1.44 [a]	34.75 ± 1.21 [a]	36.76 ± 3.74 [a]
24	20.11 ± 0.62 [a]	43.31 ± 0.69 [a]	38.52 ± 2.58 [a]	33.75 ± 1.44 [a]	34.76 ± 1.21 [a]	36.89 ± 3.80 [a]
30	20.12 ± 0.62 [a]	43.31 ± 0.69 [a]	38.53 ± 2.58 [a]	33.75 ± 1.44 [a]	34.76 ± 1.21 [a]	36.92 ± 3.81 [a]
36	20.12 ± 0.62 [a]	43.31 ± 0.69 [a]	38.53 ± 2.58 [a]	33.75 ± 1.44 [a]	34.76 ± 1.21 [a]	36.92 ± 3.81 [a]

* Means ± Standard Deviation. Means followed by the same minuscule letter within each time period did not differ by contrasts ($p > 0.05$). Analysis of variance (Treatment: $p < 0.0001$, Time: $p < 0.0001$, Time × treatment: $p < 0.0001$).

Table S2. Cumulative gas production from degradation of slow fraction (mL/g of DM) estimated by dual-pool model from samples containing increasing concentrations of BCE (mg/L) or monensin (Mon, 5 μM) along 36 h of incubation.

Time (h)	Cumulative Gas Production from Degradation of Slow Fraction (mL/g of DM) *					
	Mon	0	25	50	100	200
2	1.85 ± 0.11 [a]	5.39 ± 0.36 [a]	4.30 ± 0.85 [a]	4.55 ± 0.14 [a]	4.27 ± 0.11 [a]	3.46 ± 0.26 [a]
4	2.88 ± 0.18 [a]	7.46 ± 0.50 [b]	6.03 ± 1.18 [b,c]	6.62 ± 0.20 [b]	6.19 ± 0.162 [b,c]	4.86 ± 0.41 [c]
6	4.44 ± 0.30 [a]	10.23 ± 0.68 [b]	8.39 ± 1.61 [b,c]	9.51 ± 0.28 [b]	8.88 ± 0.24 [b,c]	6.79 ± 0.64 [c]
8	6.79 ± 0.48 [a]	13.88 ± 0.91 [b]	11.53 ± 2.18 [b,c]	13.47 ± 0.39 [b]	12.58 ± 0.35 [b]	9.40 ± 0.99 [c]
10	10.26 ± 0.78 [a]	18.54 ± 1.20 [b]	15.61 ± 2.92 [b,c]	18.70 ± 0.55 [b]	17.48 ± 0.49 [b]	12.86 ± 1.49 [a,c]
12	15.23 ± 1.17 [a]	24.30 ± 1.53 [b]	20.71 ± 3.81 [b,c]	25.29 ± 0.75 [b]	23.70 ± 0.68 [b]	17.31 ± 2.17 [c]
15	26.21 ± 1.96 [a]	34.92 ± 2.10 [b]	30.20 ± 5.45 [b,c]	37.41 ± 1.11 [b]	35.30 ± 1.05 [b]	25.95 ± 3.53 [c]
18	41.47 ± 2.75 [a,b]	47.16 ± 2.66 [a,b]	41.19 ± 7.31 [a,b]	50.84 ± 1.48 [a]	48.40 ± 1.49 [a]	36.58 ± 5.14 [b]
21	59.02 ± 3.07 [a,b]	59.55 ± 3.10 [a,b]	52.24 ± 9.16 [a,b]	63.35 ± 1.78 [a]	60.90 ± 1.93 [a,b]	48.06 ± 6.65 [b]
24	75.37 ± 2.73 [a]	70.55 ± 3.38 [a]	61.91 ± 10.77 [a]	73.28 ± 1.99 [a]	71.06 ± 2.29 [a]	58.81 ± 7.71 [a]
30	96.05 ± 1.47 [a]	85.52 ± 3.54 [a]	74.61 ± 12.93 [a]	84.61 ± 2.23 [a]	82.95 ± 2.74 [a]	74.24 ± 8.37 [a]
36	103.55 ± 1.16 [a]	92.45 ± 3.52 [a]	80.19 ± 13.91 [a]	88.77 ± 2.33 [a]	87.43 ± 2.91 [a]	81.66 ± 8.15 [a]

* Means ± Standard Deviation. Means followed by the same minuscule letter within each time period did not differ by contrasts ($p > 0.05$). Analysis of variance (Treatment: $p < 0.0001$, Time: $p < 0.0001$, Time × treatment: $p < 0.0001$).

© 2013 by the authors; licensee MDPI, Basel, Switzerland. This article is an open access article distributed under the terms and conditions of the Creative Commons Attribution license (http://creativecommons.org/licenses/by/3.0/).
